# Molecular Basis of eRF3 Recognition by the MLLE Domain of Poly(A)-Binding Protein

**DOI:** 10.1371/journal.pone.0010169

**Published:** 2010-04-14

**Authors:** Guennadi Kozlov, Kalle Gehring

**Affiliations:** 1 Department of Biochemistry, McGill University, Montréal, Québec, Canada; 2 Groupe de recherche axé sur la structure des protéines, Montréal, Québec, Canada; University of Oulu, Germany

## Abstract

PABPC1 (cytosolic poly(A)-binding protein 1) is an RNA-binding protein that binds to the poly(A) tail of mRNAs to promote translation and mRNA turnover. In addition to RNA-binding domains, PABPC1 contains a unique protein-protein interaction domain, MLLE (also known as PABC) that binds regulatory proteins and translation factors that contain a conserved 12 amino acid peptide motif termed PAM2. Eukaryotic Release Factor 3 (eRF3/GSPT1) contains two overlapping PAM2 sequences, which are required for its activity. Here, we determined the crystal structures of the MLLE domain from PABPC1 in complex with the two PAM2 regions of eRF3. The structures reveal a mechanism of cooperativity between the two PAM2 sites that increases the binding affinity but prevents the binding of more than one molecule of eRF3 to PABPC1. Relative to previous structures, the high-resolution crystal structures force a re-evaluation of the PAM2 motif and improve our understanding of the molecular basis of MLLE peptide recognition.

## Introduction

In order to rapidly respond to growth and proliferation stimuli, stress, and nutrient availability, cells use translational control as an important mechanism of gene expression. Cytoplasmic poly(A)-binding protein (PABP), also termed PABPC or PABPC1, is an essential protein that binds to and mediates the stimulatory effect of the poly(A) tail on translation initiation [1]. PABPC1 contains four N-terminal phylogenetically conserved RNA recognition motifs (RRMs) while the proline-rich, C-terminal third of the protein contains an unstructured, poorly conserved region, which harbors some protein-interaction sites [2], [3], [4], [5], [6], and a well-conserved, approximately 70-residue MLLE (*Mademoiselle*) domain, also known as PABC [7]. The MLLE domain is also found in UBR5 (or EDD), a ubiquitin-ligase [8].

Solution and crystal structures of MLLE domains from various PABPs and human UBR5 have shown that these domains consist of a bundle of 4 or 5 α-helices [7], [8], [9], [10], [11]. Previous studies showed that MLLE is a peptide-binding domain that specifically recognizes a conserved PAM2 (for PABP-interacting motif 2) sequence [12]. This PAM2 motif was initially identified in Paip1 (PABP-interacting protein 1), Paip2 and eukaryotic Release Factor 3 (eRF3) [7]. Recent crystal structures of the MLLE domain from human PABPC1 in complex with peptides from Paip2 and Ataxin-2 reveal how the most conserved elements of PAM2 motifs bind to the helices α2, α3 and α5 of MLLE but do not reveal the full range of intermolecular interactions [13], [14].

Eukaryotic release factor eRF3, also known as G_1_ to S phase transition protein GSPT, is a GTPase that facilitates the nascent peptide chain release from the terminating ribosome [15], [16], [17], [18], [19]. Two genes are known: eRF3a/GSPT1 and eRF3b/GSPT2 [20]. Structurally, both consists of a C-terminal GTPase domain, homologous to elongation factor EF1A, and an N-terminal region that contains the binding site for PABPC1 [21], [22], [23]. Unlike other PAM2-containing proteins, eRF3 possesses two overlapping PAM2 sequences (PAM2-N and PAM2-C), which each independently bind to the MLLE domain of PABPC1 with low micromolar affinity [12]. This makes them the most unusual PAM2 motifs in the family. The C-terminal site (PAM2-C), in particular, lacks a leucine recognition element that is found in essentially all other PAM2 peptides. As a pair, the two PAM2 sites bind to MLLE together with enhanced affinity and are responsible for essentially all of the free energy of binding between the intact proteins, eRF3 and PABPC1 [24].

Here, we determined two crystal structures of the MLLE domain of human PABPC1 in complex with the PAM2-N and PAM2-C peptides from human eRF3. The structures reveal a shared binding element, Phe76, that contributes to binding as part of both PAM2-N and PAM-C. The commonality between the two binding sites explains the observations that the apparent loss of a single PAM2 motif is sufficient to block eRF3 binding to PABPC1 [24], [25]. Compared to previous low resolution NMR studies, the high-resolution crystal structures reveal an enlarged lexicon of peptide recognition by the MLLE domain of PABPC1 and provide an example of binding site duplication as a means of enhancing affinity.

## Results

### Structure of the MLLE/eRF3-PAM2 complexes

In order to understand eRF3 binding by PABPC1, we crystallized the PABPC1 MLLE domain in complex with two peptides from the N-terminal portion of eRF3 that had previously been shown to mediate PABPC1 binding. We obtained well-diffracting co-crystals with eRF3 residues 67–81 (PAM2-N) and 76–90 (PAM2-C). Both crystals displayed very low solvent content resulting from tight packing. Diffraction datasets for both complexes were solved by molecular replacement and refined to 2.3 Å for PAM2-N and 1.4 Å for PAM2-C (Table 1; PDB codes 3KUI and 3KUJ).

**Table 1:** Data collection and refinement statistics.

	MLLE-eRF3 (67–81)	MLLE-eRF3 (76–90)
**Data collection**		
Space group	P2_1_2_1_2	P2_1_2_1_2
Cell dimensions		
*a*, *b*, *c* (Å)	37.43, 63.63, 32.23	45.22, 50.80, 32.12
Resolution (Å)	50-2.30 (2.34-2.30)^1^	50-1.40 (1.45-1.40)
*R*_sym_	0.094 (0.415)	0.074 (0.375)
*<I*/σ*I>*	19.3 (3.8)	19.2 (6.1)
Completeness (%)	99.7 (98.8)	99.6 (99.9)
Redundancy	7.0 (5.8)	7.5 (6.6)
Wilson B-factor (Å^2^)	47.4	14.7
**Refinement**		
Resolution (Å)	50.0-2.30	33.77-1.40
No. reflections	3559	14231
*R*_work_/*R*_free_	0.247/0.255	0.196/0.223
No. atoms		
MLLE	615	593
Peptide	92	113
Water	16	53
*B*-factors (Å^2^)		
MLLE	48.0	8.9
Peptide	45.6	14.6
Water	39.0	26.4
R.m.s deviations		
Bond lengths (Å)	0.007	0.008
Bond angles (°)	1.15	1.25
Ramachandran statistics (%)		
Most favored regions	96.2	97.4
Additional allowed regions	3.8	2.6

1 Highest resolution shell is shown in parentheses.

**Figure 1:**
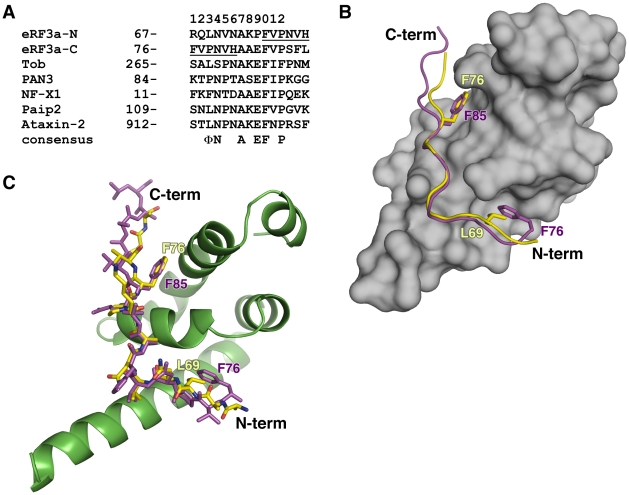
Overlapping PAM2 motifs of eRF3. (A) Sequence of MLLE-binding PAM2 motifs. PAM2-N and PAM2-C of eRF3 are aligned against sequences from Tob (transducer of Erb1), poly(A) specific ribonuclease 3 (PAN3), PABP-interacting protein 2 (Paip2) and Ataxin-2. Residues in the overlap are underlined. The consensus of most conserved residues that contribute to the MLLE/PAM2 binding is shown: Φ represents a hydrophobic residue [12]. (B) Overlaid structures of the complexes of eRF3 PAM2-N (yellow) and PAM2-C (magenta) bound to the MLLE domain (grey) from PABPC1. eRF3 Phe76 shifts position and binds to either MLLE helix α2/α3 in the PAM2-N complex or helix α3/α5 in the PAM2-C complex. Leu69 or Phe85 then occupies the vacated hydrophobic binding site. (C) Stick representation of overlaid structures of eRF3 PAM2-N (yellow) and PAM2-C (magenta) bound to the MLLE domain from PABPC1 (green).

**Figure 2:**
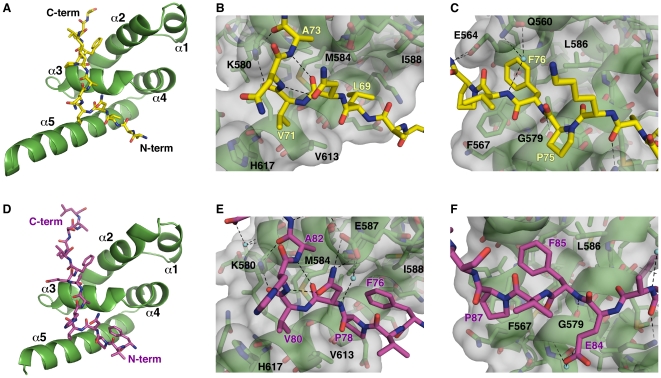
Crystal structures of the MLLE/eRF3-PAM2 complexes. (A) Cartoon representation of the MLLE domain (green) with eRF3 PAM2-N (yellow). (B) Details of the eRF3 PAM2-N structure. The eRF3 peptide makes a β-turn stabilized by hydrogen bonds between carbonyl of Asn70 and amide of Ala73 and between side chain of Asn70 and amide of Asn72. Mlle binding is mediated by several hydrophobic interactions: Leu69 binding to the pocket formed by Leu585, Ala610, and the aliphatic portion of Lys606 of MLLE, Val71 inserting into a smaller pocket formed by the side chains of Met584, Val613, Leu614 and His617, and Ala73 contacting the Met584 side chain. Intermolecular hydrogen bonds occur between the side chain of MLLE Lys580 with carbonyls of Val71 and Ala73 of Paip2. (C) C-terminal portion of PAM2-N. Phe76 binds the shallow pocket formed by Thr582, Phe567, Leu586 and Gly563 of MLLE. The amide of this phenylalanine is engaged in hydrogen bonding with carbonyl of MLLE Gly579. Pro78 of eRF3 interacts with the aromatic ring of Phe567. Side chains of Gln560 and Glu564 of MLLE form a network of intermolecular hydrogen bonds with the amides of Val77 and Asn79 of eRF3. (D) Cartoon representation of the MLLE domain (green) with eRF3 PAM2-C (magenta). (E) Details of the eRF3 PAM2-C structure. The aromatic side chain of eRF3 Phe76 binds in the hydrophobic cavity previously occupied by Leu69 of the PAM2-N peptide, while Pro78 interacts with the side chain of Val617. Side chain of Asn79 also makes a salt bridge with MLLE Glu587. (F) C-terminal portion of PAM2-C. The interactions of eRF3 Phe85 and Pro87 with MLLE are identical to those of Phe76 and Pro78 in the PAM2-N structure. The side chain of Glu84 of eRF3 forms intermolecular hydrogen bonds with amides of Gly579 and Lys580 via an ordered water molecule. Hydrogen bonds are shown in black, ordered water molecules in cyan. Figures were made with PyMOL (http://pymol.sourceforge.net/).

Overlay of the MLLE/PAM2-N and MLLE/PAM2-C structures reveals striking similarity of the bound conformations despite the highly divergent peptide sequences (Fig. 1). In both structures, the peptide binds by wrapping around the highly conserved KITGMLLE signature motif of MLLE and interacting with the hydrophobic pockets between helices α2 and α3 and between helices α3 and α5 of MLLE. The peptides adopt an extended conformation interrupted by a β-turn at residues Asn70-Val71-Asn72 in PAM2-N and at residues Asn79-Val80-His81 in PAM2-C (Fig. 2).

The MLLE/PAM2 interactions are mostly hydrophobic with as many as five conserved recognition elements. In the N-terminal part of the peptide, the side chain of eRF3 Leu69 of PAM2-N inserts into a hydrophobic pocket formed by the side chains of Met584, Ile588, Ala610 and the aliphatic parts of Lys606 and Glu609 (Fig. 2B). This same pocket is occupied by Phe76 and Pro78 in the PAM2-C structure (Fig. 2 E). At the tip of the peptide β-turn, the side chain of Val71/Val80 makes an additional hydrophobic contact with the MLLE side chains of Val613 and His617. Another hydrophobic interaction involves the invariantly conserved alanine at residue 73 of PAM2-N and at 82 of PAM2-C. At the C-terminus of both peptides, Phe76/Phe85 fits into the shallow cavity formed by Gly563, the methyl groups of Thr582 and Leu586 and the aliphatic part of Glu564 (Fig. 2C, 2F). Mutagenesis studies with Paip2 have shown that this is the single most important determinant of binding [12]. In addition to its essential side chain, the amide of Phe76/Phe85 hydrogen bonds with the carbonyl of Gly579. This close approach of the two backbones is reflected in the perfect conservation of this glycine in the KITGMLLE motif.

The most significant differences between the two peptide complexes occur at the N-terminus where PAM2-C lacks a highly conserved leucine residue. Mutagenesis of this residue in the PAM2 motif of Paip2 led to a loss of three orders of magnitude of binding affinity [12]. In its place, Phe76 bends back to partly occupy the space vacated by the missing leucine side chain. Thus eRF3 Phe76 plays two distinct roles in the complexes, alternately occupying the hydrophobic pocket between MLLE helices α2 and α3 in the PAM2-N complex and between helices α3 and α5 in the PAM2-C complex (Fig. 1B). The simultaneous requirement for Phe76 for MLLE binding by both PAM2-N and PAM2-C explains the previous observations of a one-to-one stoichiometry of eRF3 binding to PABPC1 despite the existence of two PAM2 motifs [24], [26].

### Sequence conservation in PAM2 motifs

The high resolution structures of the peptides bound to the PABPC1 MLLE domain allows a re-evaluation of the definition of the PAM2 motif and the residues that are most essential for binding [13], [14]. We had previously described the motif as comprising 12 residues with a highly conserved leucine residue at position 3, alanine at position 7 and phenylalanine at position 10 [7], [12]. In light of the eRF3 structures, the refined definition is --Φ-(P/V)-A--F-P, where Φ, at position 3, is a hydrophobic residue, usually leucine but occasionally proline or phenylalanine (Fig. 1A). Positions 1 and 2 are normally occupied by polar or charged residues, which do not participate in MLLE binding except in the MLLE/PAM2-C structure where phenylalanine in position 1 binds in combination with proline at position 3. Position 4 is a polar residue, usually asparagine or less often serine, whose side chain makes an intermolecular ionic contact with Glu587 (the “E” in MLLE) and also stabilizes the β-turn via a hydrogen bond with the amide of residue at position 6 (Fig. 2B and 2E). The side chain of proline, valine or occasionally threonine in position 5 fits snugly into a small hydrophobic cavity in helix α5 of MLLE. The common occurrence of proline in this position may due to its propensity to form β-turns. Its mutagenesis to alanine decreased the affinity of the PAM2 motif of Paip2 by 5-fold [12]. While positions 6 and 8 are solvent-exposed and do not to contribute to MLLE binding, position 7 is invariantly alanine and binds to the methionine of the MLLE signature motif. Glutamic acid is preferred at position 9 and makes hydrogen bonds with amides of Gly579 and Lys580 in the PAM2-C structure via ordered water molecules (Fig. 2F). As previously noted, position 10 is invariantly phenylalanine and the single most important residue for binding. While position 11 is not conserved, position 12 is usually occupied by proline, which makes an addition contact with MLLE domain (Fig. 2C and 2E). While there is no sequence conservation beyond position 12, the MLLE-binding region extends by at least one more residue, as the backbone amide at position 13 makes a hydrogen bond with the side chain of conserved Glu564 of MLLE. Interestingly, most intermolecular polar contacts involve the backbone of the PAM2 peptide and do not directly affect side chain specificity.

## Discussion

eRF3 is the only known protein with two high affinity PAM2 sites and the only example of overlapping sites. What is the function of the juxtaposed sites? Previous studies showed that peptides containing the individual PAM2 sites display moderate binding affinities (K_d_ of 3.9 and 3.1 µM) while the peptide containing the overlapping site shows a three-fold increase in binding affinity (1.3 µM) [12]. This is 1.3 times higher than the value calculated for two independent sites, based on the law of mass action, and reflects a small degree of cooperativity between the sites. In agreement with this, NMR titrations showed a large amount of conformational exchange occurs between the two sites, presumably as the MLLE domain slides along the eRF3 peptide binding alternately to PAM2-N and PAM2-C [12]. One important distinction is that the eRF3-PABPC1 complex exists exclusively in one-to-one stoichiometry as Phe76 binding is essential for both complexes (Fig. 1B). Thus the addition of a second, overlapping PAM2 site acts to augment the affinity of the eRF3-PABPC1 interaction without changing the stoichiometry of the interaction. The second site also provides redundancy and reduces the frequency of loss of binding due to a mutation in one of the PAM2 sites.

Intriguingly, this redundancy and higher affinity is lost in a fraction of eRF3 in cells that is proteolytically cleaved in the middle of PAM2-N site leaving Ala73 as the N-terminal residue [27]. This cleavage product retains an intact PAM2-C site and, based on our crystal structure and previous peptide affinity measurements, would be expected to interact with PABPC1 with 3-fold lower affinity. The protease responsible for the cleavage of eRF3 is not known but the cleavage is thought to be a regulatory mechanism to regulate eRF3 levels/localization and potentiates apoptosis by liberating caspases from inhibitors of apoptosis (IAPs) that bind to the cleaved form of eRF3.

**Figure 3:**
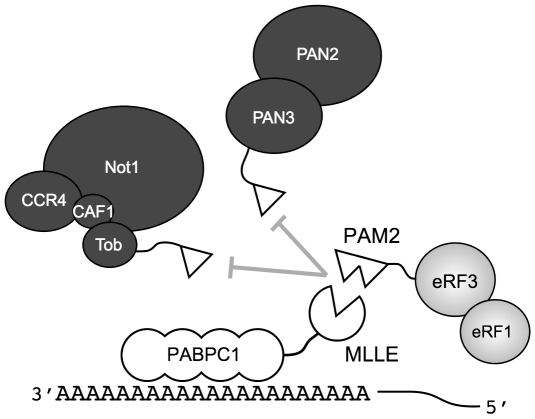
Model of PABPC1 interacting with proteins in translation termination and deadenylation. The mRNA poly(A) binds multiple PABPC1 proteins through the N-terminal RRM1-4 domains. The C-terminal MLLE domain of PABPC1 (open circle missing a triangle) binds proteins containing PAM2 motifs (open triangles). The higher affinity PABPC1-eRF3 interaction inhibits the recruitment of two mRNA deadenylation complexes, PAN2/3 and Ccr4-Not-Caf1, to the mRNA. These complexes bind the MLLE binding via the PAM2 motifs of PAN3 and Tob, a member of the antiproliferative BTG family. The MLLE-PAM2 interaction is required for PABPC1-dependent stimulation of deadenylase activity by both PAN3 and Tob [26], [31].

The eRF3-PABPC1 interaction is central to eRF3′s function in regulating mRNA deadenylation [26]. Through the MLLE domain, PABPC1 interacts with two mRNA deadenylation systems, PAN2-PAN3 and Ccr4-Not-Caf1, and contributes to deadenylation of cytosolic mRNAs (Fig. 3). In both systems, binding occurs through a linker protein, PAN3 or Tob, that contains a PAM2 motif [28], [29], [30], [31]. These deadenylase complexes compete with eRF3 for binding to the MLLE domain so that their recruitment to the mRNA is regulated by eRF3 [26]. Peptide binding studies show that the overlapped PAM2 motifs of eRF3 binding with significantly (up to 30 fold) higher affinity than the PAM2 motifs of PAN3 and Tob [12], [28], [32]. Comparison of the PAM2 motifs of PAN3 and eRF3 highlights the importance of the N-terminal residues (Fig. 1A). While PAN3 does have a proline residue at position 3, it lacks the phenylalanine residue at position 1 that binds to the MLLE hydrophobic pocket between helices α3 and α5.

Binding studies with the full-length proteins PABPC1 and eRF3 have reported affinities very similar to those measured for MLLE binding the overlapping PAM2 site [24]. Thus, it appears that the PAM2 motifs of eRF3 are the only interaction sites for binding to PABPC1. This is supported by mutagenesis studies. Recently, a four residue mutant of eRF3 (L69K, N72A, A73K, F76A) was shown to have a compromised binding to PABPC1 [25]. Similarly, mutagenesis of the PAM2-C sequence (H81A, A82K, F85A) was shown to block eRF3 binding to PABPC1 as measured by isothermal titration calorimetry [24]. These are consistent with studies of other PAM2-containing proteins, which have shown that the phenylalanine residue at position 10 is required for binding and function. Specifically, mutagenesis of PAN3 (F93A) [28], Tob (F274A) [26] and NFX1-123 (F20A) [33] were all shown to disrupt binding to PABPC1 in cells.

In summary, the high-resolution X-ray crystal structures presented here significantly improve our understanding of the eRF3-PABPC1 interactions and, in general, PAM2 recognition by the MLLE domain of PABPC1. Despite significant sequence divergence, the two PAM2 peptides of eRF3 bind with almost identical conformations and show synergy when combined as an overlapped pair of binding sites. The refined definition of the PAM2 motif strongly suggests that other more divergent PAM2-containing proteins, such as PAN3 and Tob, will interact with PABPC1 in a manner very similar to the eRF3 PAM2 motifs.

## Materials and Methods

### MLLE expression, purification and peptide synthesis

The MLLE domain (residues 544–626) of human PABPC1 was cloned into pGEX-6P-1 vector (Amersham-Pharmacia) and expressed and purified as described previously for the longer 544–636 fragment [12] with the addition of a final size-exclusion chromatography step.

The PAM2 peptides were synthesized by Fmoc solid-phase peptide synthesis and purified by reverse phase chromatography on a C18 column (Vydac, Hesperia, CA). The composition and purity of the peptides was verified by ion-spray quadruple mass spectroscopy.

### Crystallization

Crystallization conditions for the MLLE domain in complex with the PAM2 peptides from eRF3 were identified utilizing hanging drop vapor diffusion with the AmSO4 crystallization suite (QIAGEN). The best MLLE/PAM2-N crystals were obtained by equilibrating a 1 µl drop of PABPC1 (544–626)/eRF3 (67–81) mixture (10 mg/ml) in 1∶2 molar ratio in a buffer (10 mM MES, 100 mM NaCl, pH 6.3), mixed with 1 µl of reservoir solution containing 2.1 M ammonium sulfate, 0.2 M sodium sulfate, 10 mM zinc chloride and 0.1 M sodium acetate at pH 5.4. Crystals grew in 10–20 days at 22°C. The solution for cryoprotection contained the reservoir solution with the addition of 15% (v/v) glycerol. The crystals contain one MLLE and one PAM2-N molecule in the asymmetric unit corresponding to V_m_ = 1.7 Å^3^ Da^−1^ and a solvent content of 28.5%. The best MLLE/PAM2-C crystals were obtained by equilibrating a 1 µl drop of PABPC1 (544–626)/eRF3 (76–90) mixture (10 mg/ml) in 1∶2 molar ratio in a buffer (10 mM MES, 100 mM NaCl, pH 6.3), mixed with 1 µl of reservoir solution containing 1.4 M ammonium sulfate and 0.1 M citric acid pH 4.0. Crystals grew in 5–10 days at 22°C. The solution for cryoprotection contained the reservoir solution with the addition of 20% (v/v) glycerol. The crystals contain one MLLE and one PAM2-C molecule in the asymmetric unit corresponding to V_m_ = 1.7 Å^3^ Da^−1^ and a solvent content of 26.1% [34].

### Structure solution and refinement

Diffraction data from a single crystal of the MLLE/PAM2-C complex were collected on an ADSC Quantum-210 CCD detector (Area Detector Systems Corp.) at beamline F2 at the Cornell High-Energy Synchrotron Source (CHESS) (Table 1). Diffraction data from a single crystal of the MLLE/PAM2-N complex were collected on a Rigaku R-Axis IV++ imaging-plate detector at the McGill Macromolecular X-ray Diffraction Facility. Data processing and scaling were performed with HKL2000 [35]. The structures were determined by molecular replacement with Phaser [36], using the coordinates of unliganded MLLE from human EDD (PDB entry 1I2T). The initial MLLE/PAM2-C model obtained from Phaser was completed and adjusted with the program Coot [37] and improved by several cycles of refinement, using the program REFMAC 5.2 [38] and model refitting. At the latest stage of refinement, we also applied the translation-libration-screw (TLS) option [39] with final density for PABPC1 residues 544–620 and eRF3 residues 76–90. The MLLE/PAM2-N model was refined using CNS [40] with density for PABPC1 residues 545–625 and eRF3 residues 67–80. The models have good stereochemistry according to the program PROCHECK [41] (Table 1) and well-defined density for the bound peptide (Supplemental Figure S1).

## Supporting Information

Figure S1 Electron density from the eRF3 PAM2-N (A) and PAM2-C (B) peptides contoured at 1 σ from the 2FO-FC omit maps. The PAM2-N (yellow) and PAM2-C (magenta) peptides are shown in stick representation and the MLLE domain is shown in cartoon representation (green).
(0.15 MB PDF)
