# Supplementary material for: Molecular Basis of eRF3 Recognition by the MLLE Domain of Poly(A)-Binding Protein
Source: PLoS One. 2010 Apr 14;5(4):e10169. doi: 10.1371/journal.pone.0010169 (PMC2854688; doi:10.1371/journal.pone.0010169)
Supplement: Figure S1 — Electron density from the eRF3 PAM2-N (A) and PAM2-C (B) peptides contoured at 1 σ from the 2FO-FC omit maps. The PAM2-N (yellow) and PAM2-C (magenta) peptides are shown in stick representation and the MLLE domain is shown in cartoon representation (green). (0.15 MB PDF) [file pone.0010169.s001.pdf]

A

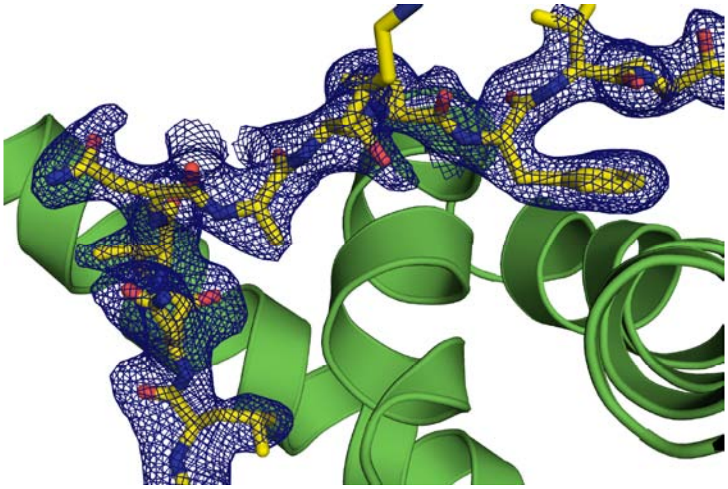

B

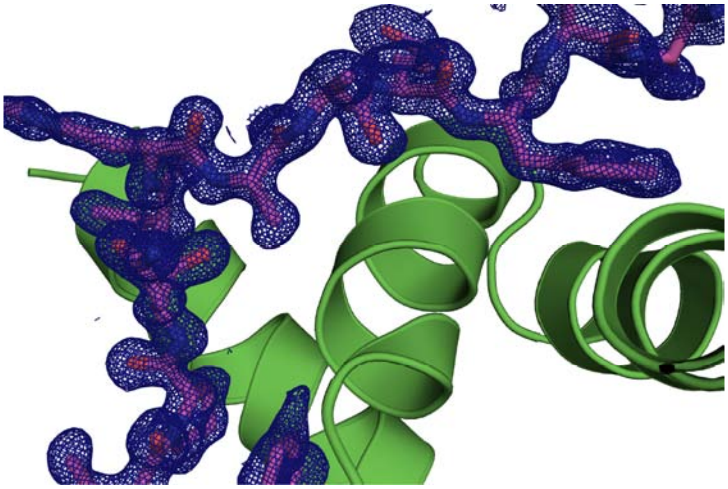

Supplemental Figure 1. Electron density from the eRF3 PAM2-N (A) and PAM2-C (B) peptides contoured at 1 σ from the $2F_O$-F_C omit maps. The PAM2-N (yellow) and PAM2-C (magenta) peptides are shown in stick representation and the MLLE domain is shown in cartoon representation (green).
